# Holistic Governance for Sustainable Public Services: Reshaping Government–Enterprise Relationships in China’s Digital Government Context

**DOI:** 10.3390/ijerph17051778

**Published:** 2020-03-09

**Authors:** Xuesong Li, Yunlong Ding

**Affiliations:** School of Management, Harbin Institute of Technology, Harbin 150001, China; 17b910061@stu.hit.edu.cn

**Keywords:** holistic governance, sustainable public services, digital government, government–enterprise relationships

## Abstract

The notion of holistic governance was originally proposed to make up for the fragmentation of public service provision. However, such a notion also has a great potential to be transferred and understood in the digital government context in China, where there is an increasing need to reshape the landscape of government–enterprise relationships that can enable enterprises to involvement effectively in holistic governance, or the planning and design of public services. However, previous empirical studies on holistic governance have neglected the question of how to make this happen. The aim of this article is to fill these gaps, building on holistic governance theory, this article offers a theoretical framework for government–enterprise relationships under the holistic governance paradigm. The framework identifies a comprehensive set of relationships that explain how these relationships affect enterprises’ participation in public service provision. The empirical analysis is based on case studies of four e-services cooperation programs in China. We report three main findings. First, economic incentive should be developed in combination with a holistic governance strategy in order to encourage policymakers to reshape government–enterprise relationships. Second, it seems that the implementation of holistic governance is more effective when complemented with a managerial strategy in relation to organizational transformation. Finally, trust-building between governments and enterprises plays a pivotal role in nurturing the holistic governance paradigm. These findings have important policy implications for efforts to promote enterprise participation and cross-sector solutions to fragmented public service provision.

## 1. Introduction

In the pursuit of sustainable public service, i.e., “public services should be efficient, effective, economical and equitable” [1], increasingly complicated public issues and the drastic advancement of modern information and communication technology (ICT) have placed high demands on local governments which are supposed to address in a coordinated and agile way a multitude of social, economic, cultural and other policy challenges [2,3]. Currently, governments are struggling to meet such challenges, and are forced to rely on limited capacity and fragmented authority to deal with policy challenges embedded in their indigenous governance contexts. Confronted with such challenges, governments have increasingly used indirect management tools and co-production to deliver public services, especially with regard to the implementation of public e-services projects [4]. Enterprises or Internet companies receive increasing attention in public administration research because of their prominent role in the provision and production of public services [5]. As a result, there is a growing trend that governments provide public services through new interactive relationships with enterprises. These relationships are characterized by the spontaneous alliance of fragmented enterprises and governments into a holistic service delivery system [6]. In such a novel system, established governance processes for strengthening regulation and supervision are no longer suitable for meeting the requirements of sustainable public service. Governments are thus expected to adopt a new governance paradigm to accommodate the evolving and dynamic working relationships with enterprises.

Notably, the expectation towards sustainable public services is actually shifting attention away from organizational institutions of governance to the service processes of governance [7]. This emphasis on implementing non-fragmented arrangements that can adapt to diversified service needs in their indigenous social, economic, and other contexts is echoed in the principles of holistic governance. It is also symbolic of the progression of the service delivery system from fragmentation to integration to coordination, with a profound impact on government–enterprise relationships, i.e., on “multiple pathways in which government and enterprise interact” [8]. The concept of holistic governance has been formulated in studies related to national public service systems but possesses the practical potential to be applied to multiple institutional arrangements. With the help of this concept, holistic governance covers “vertical and horizontal modes of public affairs governance” that has increased private actors’ participation in order to achieve a public purpose that could be used to achieve the co-production of public services [9]. Recently, there have been calls to apply the perspective of holistic governance to the process of carrying out public e-services projects [10]. The key feature of the holistic governance paradigm consists in laying emphasis on the unique role of governments which provide information, data, aggregation processes, and other policy tools in an attempt to empower enterprises to deliver public services [11]. Despite the fact that the view of holistic governance is potentially suitable to capture the need of governments to establish governance practices in response to sustainable public service at a conceptual level, it still needs to be further extended and empirically tested in the digital government context.

Digital government has been seen as a source of critical factors, benchmarking and analytical frameworks to evaluate public services and the impact of technologies on service production and provision. The increasing interest of the digital government in public service theory is a response to the difficulties in fulfilling citizens’ expectations in public service delivery following the lack of success of new public management reforms [12]. As a kind of technological change, digital government driven by information technology can significantly improve the sustainability of government departments. Sustainability not only means the technological transformation of internal government organization but also the ideological transformation of external public service supply mode [13,14]. Given the background of China’s digital government, the supply of public services depends more on co-production with third parties (especially Internet enterprises) [15]. The digital technology transforms the supply mode of public services from the single-agent supply of the government to the multi-agent supply. To find a balance between maintaining stability control and achieving dynamic flexibility, governments engaged in digital government–enterprise cooperation projects are required to break through the limitation of traditional bureaucratic governance structure and rethink the way empowerment and orchestration are distributed among governments and enterprises. During the process, governments cannot just play the role of central authority but respect the autonomy and self-control of stakeholders. In consequence, the change in this role leads to the transformation of the relationship between governments and enterprises from vertical control to horizontal coordination [16].

Existing research on public administration focuses on the instrumental orientations of enterprises involved in the delivery of public services [17]. **The key question is about how to regulate relationships between governments and enterprises so that enterprises can serve as a governance tool for the government to produce and provide public e-services more effectively**. The role played by enterprises in designing, planning, and innovating public e-services remains a blind spot in the policy science and public administration scholarship despite the fact that enterprises have been playing an important role. Even worse, this limitation is further worsening when governments at all levels are currently facing operational fragmentation and relying increasingly on enterprises for linking fragmented public service system in China. A more comprehensive understanding of the characteristics of relationships between governments and enterprises toward holistic governance or public service supply in China is, thus, desperately needed. Informed by literature concerning co-production, holistic governance, and relationships between governments and enterprises, the aim of this article is to offer a comprehensive theoretical framework for government–enterprise relationships under the holistic governance paradigm based on the analysis of four public e-services cases of government–enterprise cooperation in public e-services projects in China. More specifically, case analysis is used to discuss relationships between governments and enterprises in terms of holistic governance, and how these relationships affect enterprises’ participation in public service provision. Theoretically and practically, several suggestions provided by the results based on digital practices in China are relevant to researchers and policymakers interested in utilizing holistic governance to facilitate sustainable public services.

The article is structured as follows. In Section 2, we discuss existing research on the governance of collaboration between government and non-government actors, and the emergence of the concept of holistic governance in the digital government context. In Section 3, we explain four cases of information technology (IT)-related project collaboration between government and non-government actors in China as our sources of empirical data and illustrate the methods of data collection. In Section 4, we present the findings from the analysis of the four cases. In Section 5, we present the implications of our study for both the research and practice of holistic governance in China’s digital government context and discuss the limitations of the study. In Section 6, we identify avenues for future research.

## 2. Background

### 2.1. Holistic Governance in the Digital Government Context

Within public administration literature, holistic governance serves as an important theoretical framework for understanding enterprises’ involvement in terms of public service supply [18,19]. Enterprises’ involvement in terms of public service provision, according to the theory of holistic governance, makes it possible to improve the cost-effectiveness and quality of public services [20]. Traced back to the “White Paper on Modern Government Policy” issued by the British government in 1997, the concept of “holistic governance” was coined to explain a new governance philosophy to resolve the fragmentation of services and the complexity stemming from intricate policy challenges such as poverty alleviation, trans-boundary environmental pollution, digital divide and e-waste [21]. Moreover, holistic governance was used by the British government to reflect the fact that the value of public services cannot fully be captured without proactive coordination and active involvement of the service receiver. Originally speaking, public administration scholarships have pointed out that it is possible for both individual citizens and non-governmental organizations (NGOs) to get involved in this type of holistic governance of public services. For example, Rummery [22] illustrated the concept of holistic governance based on the concerted effort of enterprises and governance in improving educational services. Janssen and Klievink [23] discussed the importance of collective forms of holistic governance, further pointing out that holism requires consideration from different perspectives including holistic working culture, integrated information systems, dialogue between government, enterprises, and citizens. A recent review of holistic governance shows that Li et al. [24] typically illustrated the form of holistic governance by using the example of local government departments that work with enterprises to support low-carbon strategies in a pilot area, thus providing social benefits for national low-carbon development. Social media platforms and listed enterprises often play an instrumental role in facilitating and organizing citizens’ involvement in environmental protection activities [25,26,27].

Since holistic governance in the digital government context is vaguely referred to as “one idea is to solve fragmentation problems of governmental operation” [28], it has not been clearly defined. Nevertheless, from the perspective of government governance, four key characteristics of holistic governance in the digital government context have been identified: “Cross-boundary aggregation of information resources, decision-making sharing between government and enterprise, efforts to mobilize internal and external capabilities, simultaneous activation at various jurisdictional levels” [29,30]. However, there is no denying that the theoretical perspectives of holistic governance should be practically tested in the context of China’s digital government so that further research is required to identify the manifestation of the government–enterprise relationships under the key characteristics of holistic governance.

### 2.2. Reshaping Government–Enterprise Relationships Underpins Holistic Governance

In the literature on political science and public administration, the study that is concerned with government–enterprise relationships in maintaining holistic governance focuses on the exchange or reshaping of what roles enterprise and government play in the course of governance [31,32,33,34]. The rapid rise of private enterprise further promotes the formation of holistic governance in the digital era. Consequently, the concept of holistic governance is extended to the coordination and aggregation of capabilities, knowledge, and resources between the government and enterprises. The emergence of influential social media platforms such as QQ, Weibo and WeChat, and of innovative e-commerce platforms such as Jingdong and Taobao, are representative of a burgeoning digital ecosystem that governments have to make an adaptive response to when formulating service policies and new modes of cooperation with digital private enterprises [35]. The latest sign is that the Chinese government has opened up the public e-services market to create structural opportunities for private elite enterprise to participate [36].

Considering the Chinese context, some general attributes of the role that reshaping government–enterprise relationships play in maintaining holistic governance may be anticipated. Initially, the major task of reshaping government–enterprise relationships is to convince the central government of policy changes. As China is a multi-tiered system of government, in order to ensure their business activities can be carried out nationwide, enterprises are equipped with sufficient incentives to influence the decision-making of the central government across these layers. More concretely, to change the local policy environment, enterprises try to convince the central government of paying attention to local practices, developing advanced experience and understanding the market demand for policy changes [37]. It is apparent that the legitimacy of relationships for sustainable public services would benefit from clearer linkage to existing institutions and multilateral agreements [38]. For the reason that political representation of national social-ecology is still monopolized by the state, the aggressive policy activities conducted by the private enterprises often lack the support from formal public institutions and tend to expand business rather than supplement policy [39]. The evolving political system has provided access to private enterprises, such as empowering private entrepreneurs as members of the parliament, which attracts private entrepreneurs to the political process and maximizes their business interests [40]. In the case of local governance arrangement for sustainable public services, legitimating the reshaping of government–enterprise relationships is justified through the governance forms of common interests [41,42].

## 3. Conceptual Framework and Method

### 3.1. Conceptual Framework

Although scholars have discussed holistic governance’s implications for sustainable public services over the past decades, the term holistic governance for sustainable public services remains nascent in its conceptualization. More specifically, Leat [43] introduced the term primarily to describe the governmental governance structure in which citizens become more involved. Despite that insightful prospect, holistic governance tended not only to provide more opportunities for citizen engagement but also to place a heavier weight on the role of citizens than that of enterprises, especially the rising Internet enterprises, in the digital government context [44]. Besides, some have suggested that conceptual frameworks related to holistic governance entailing democratic attributes in governance, such as collaborative, monitorial and deliberative processes are available [45,46]. However, these works, though grasping macroscopical and important aspects of holistic governance, such as openness and public participation, tended to ignore a microcosmic actor’s relational aspects of holistic governance, leading to the lack of effective guidance in the holistic governance practices of the government [47]. Therefore, in this paper, holistic governance for sustainable public services is conceptualized as a mechanism in which the government and enterprises interact with each other continuously on the technology-mediated platform in the process of developing policies and of addressing problems during public service delivery.

**Figure 1:**
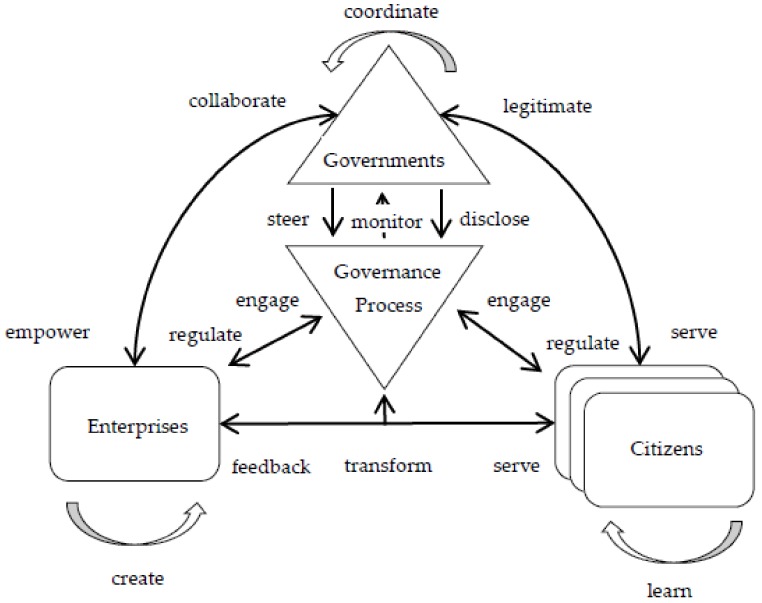
Holistic governance framework for sustainable public service.

Janowski et al. [48] develop a conceptual framework for citizen–administration relationships under the platform governance paradigm, which is used to explain how the government can empower citizens to create value by themselves. This study is based on an international perspective, which is difficult to apply directly to the Chinese context. China’s governments are more willing to create public value in the form of third-party (including enterprise and citizen) cooperation than the full authorization of citizens. On the basis of this research, we develop an extended framework to explain the relationship between government, enterprise, and citizen under the holistic governance paradigm, and introduce a processing element, which is an institutional arrangement in which three subjects jointly create public value. The framework, the holistic governance for sustainable public service, is depicted in Figure 1.

Based on holistic governance theory, the article analyzes the conditions which can contribute to spreading holistic governance along with the whole range of actors occurring in the cycle of public services in a digital government context. In particular, we probed into the roles played by diverse actors, interactive relationship, the organizational, managerial as well as institutional issues which can support the adoption of holistic governance. Furthermore, holistic governance theory is applied to show the positive impact exerted by the opportunity of creating synergies between government and enterprise on public value. More specifically, an integrative framework combining three conceptual elements, namely, four main entities as the actor, governance as a process, and various relationships between them are illustrated.

The framework contains four main entities. The first, *Governments*, represent all levels of governments that are endowed with authorization and mandate to regulate other actors and can steer and disclose the governance process. The second, *Enterprises*, stands for the private enterprise associated with public e-services delivery. These *Enterprises* often predominate in social, electronic payment, e-commerce, etc., and have the ability to replace the government’s independent provider of public services in this area. In the governance process, *Governments* delegate powers to *Enterprises* to act on their behalf, co-design, support technology and participate in service processes. The third, *Citizens*, are representative of the needs and outcomes of sustainable public services and comprise *Enterprises*, *Citizens* and other non-state actors that benefit from the holistic governance arrangement. The last, *Governance Process*, is symbolic of the concrete arrangement that provides public services, and sketches the interactions between *Governments*, *Enterprises* and *Citizens*, political representatives, and technological intermediary, providing a special intermediary mechanism of *Enterprises* and *Citizens*’ opportunities to influence and participate in policy-making and service processes. *Governance Process* takes more pluralistic patterns of rules than do policy tools, putting more emphasis on the process.

The framework contains 14 government–enterprise relationships. The categorization and definition are depicted in Table 1. In order to accurately investigate and define the characteristics of government–enterprise relations under holistic governance, a literature analysis was conducted in this article. The literature search was carried out on the Scopus, Elsevier database using the family of search terms: “sustainable public services” AND (“governance” OR ” holistic governance”). Altogether, 14 government–enterprise relationships were uncovered in the process:

These relationships are mapped into holistic governance paradigms and integrated into conceptual framework of holistic governance for sustainable public services. The internal behavioral consistency at all levels of government is represented by the *coordinate* relationship. Government enacts governance process through the *steer* relationship, and in the meantime authorizes enterprise to provide public services to citizen through the *empower* relationship. Moreover, government and enterprise deliver public services to citizen by means of the *serve* relationship. As for citizens, they can also provide direct feedback to enterprise through the *feedback* relationship. In return, citizen and enterprise engage in the governance process through the *engage* relationship. Dependent on this interaction, citizen and enterprise are thus able to supervise government governance processes through the *monitor* relationship, and can impose direct influence on the transformation of government via the *transform* relationship. In addition, they can also legitimize government to act on their behalf through the *legitimize* relationship. By virtue of the governance process, it is likely that government can open its decisions and actions to enterprise and citizen in order to build trust as part of the *disclose* relationship. In turn, enterprises can share knowledge and access political resources with the government, which comprises part of the *collaborate* relationship. Thanks to such collaboration, enterprises can thus develop themselves through the *create* relationship. Meanwhile, citizens can co-create public value and development futures as part of the *learn* relationship.

### 3.2. Method

The research method of this article is the case study. We applied the conceptual framework to analyze four Chinese case studies. The analysis captures the presence of government–enterprise relationships and identifies varieties of holistic governance for sustainable public services present among the cases. We selected the four cases based on the four key characteristics of holistic governance in the digital government context. We operationalized each characteristic as follows: *cross-boundary aggregation of information resources* as the presence of integration of government and enterprise service processes, data and other information resources; *decision-making sharing between government and enterprise* as the presence of multi-centricity of decision-making authorities and the presence of decision-making processes that do not follow a hierarchical order; efforts to *mobilize internal and external capabilities* as the presence of complementarity of superior resources (e.g., technology, budget, human resources) as well as the presence of exchange of knowledge of project participants; *simultaneous activation at various jurisdictional levels* as the presence of vertical integration of government structure to enhance intergovernmental policymaking and implementation during the development of the project.

Multiple sources of secondary data are collected for the analysis of the roles of reshaping government–enterprise relationships in maintaining holistic governance. Additionally, official documents and media reports about public e-services cooperation projects were obtained from publicly accessible channels like Internet-based media, government webpages, and online databases. Notably, the focus lies in the interactions of Internet enterprises with multiple levels of governments between 2016 and 2019. All the case studies are listed in Table 2.

## 4. Case Study Analysis

### 4.1. Case 1—China State Council App Project

#### 4.1.1. Overview of Project

The Chinese Government Network Operation Center has set up a new technical team to redesign the State Council App which has been added with important news, premier, policies, departments, localities, services, inspection, and other columns at present. It will release the state council’s major decisions and arrangements, important policy documents, important meetings of state council leaders, inspection visits and other government affairs information. On the basis of strengthening the function of information release, this app has launched digital functions such as policy search, data query, and customized services. Meanwhile, the core innovation of the State Council App lies in rendering services related to government business to society and building an information release system based on social media. This system is a fanned information release structure with the State Council App as the information release center and Weibo, WeChat, QQ and other social media as the diffusion channels. These social media buttons are provided on the homepage of each item of government news so that users can directly re-post the news to social media by clicking on the buttons. Internet companies enjoy the advantage of a large group of Internet users so that they can raise support from government and citizens in multiple natural ways. An impressive case was that in early 2017, the State Council released a notice on holiday arrangements on its app, which received more than 50 million online readings in 10 min after being forwarded by social media. In addition, the State Council App has added an online evaluation system, making it possible for citizens to directly make suggestions and comments on policy news. In this way, valuable suggestions can be adopted by the State Council to facilitate the formulation of new policies.

#### 4.1.2. Framework Instantiation

**Figure 2:**
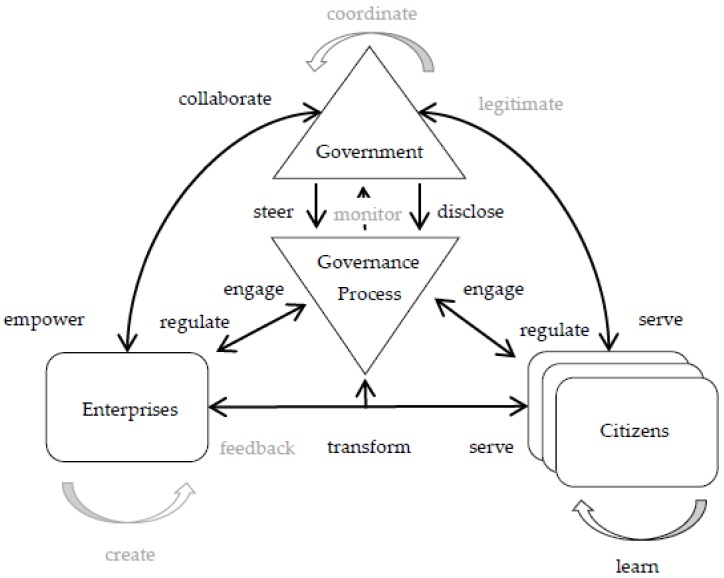
Framework instantiation, China State Council App project case.

The instantiation of the framework in this case is depicted in Figure 2. The State Council is represented as *Government* whereas Internet companies are represented as *Enterprise*, and users participating in the information release system are represented as *Citizens*. Developed by government through the *steer* relationship, the governance process includes the concept of development and application of open processes and disclosure policies. The new role of government concerning the supply of open processes and disclosure policies is implemented through *serve* and *empower* relationships; while its authorization role is manifested through the *regulate* relationship. The coordinating role of enterprises is played through *mediate* and *serve* relationships. Additionally, the information release system calls enterprise and citizen to collaborate by sharing knowledge and sources in the policy-making process through *transform* and *learn* relationships, respectively.

### 4.2. Case 2—The Guizhou-Cloud Big Data Project

#### 4.2.1. Overview of Project

The Guizhou province has set up “Big Data Industry Development Leading Group (BDILDLG)” with the governor in charge, aiming to lay an organizational foundation for the implementation of big data strategy in 2014. In August of the same year, BDILDLG was in a big data service contract with the Alibaba Cloud Computing Co. LTD. (Beijing, China), mainly dedicated to developing a provincial-level government data cloud platform and the IT infrastructure of the Guizhou provincial authority. In October 2014, the “Guizhou-cloud Government Affairs System” was officially launched, whose establishment has thus promoted the aggregation of data resources of the municipal, district and county government departments, and broken the fragmentation of departments with the help of big data technology. Guizhou province is considered a natural data storage center owing to its cold weather, stable geological structure and low electricity price, which are conducive to the heat dissipation and storage of IT equipment. It is also because of these merits that Guizhou province has established the “Guizhou Big Data Industry Development Co., Ltd. (Guiyang, China)” in November 2014, which is controlled by the government. The company’s main business is to set up and operate data storage centers. Thanks to the policy support from Guizhou province, Guizhou-cloud company has signed strategic cooperation framework agreements with Alibaba, Tencent, Qualcomm, Didi, Apple, Jingdong and other tech giants, providing cloud services for data development, storage, and processing for these companies. It is noteworthy that since 2018, Guizhou-cloud company has been authorized as the sole partner of U. S. technology giant Apple. in China to operate cloud services, and is solely responsible for the operation of iCloud in mainland China. With the assistance of the star effect of strategic cooperation with Apple., the Guizhou-cloud big data project has attracted a large number of Internet companies to join, forming China’s first big data industry cluster, which has promoted the rapid development of the regional economy.

#### 4.2.2. Framework Instantiation

**Figure 3:**
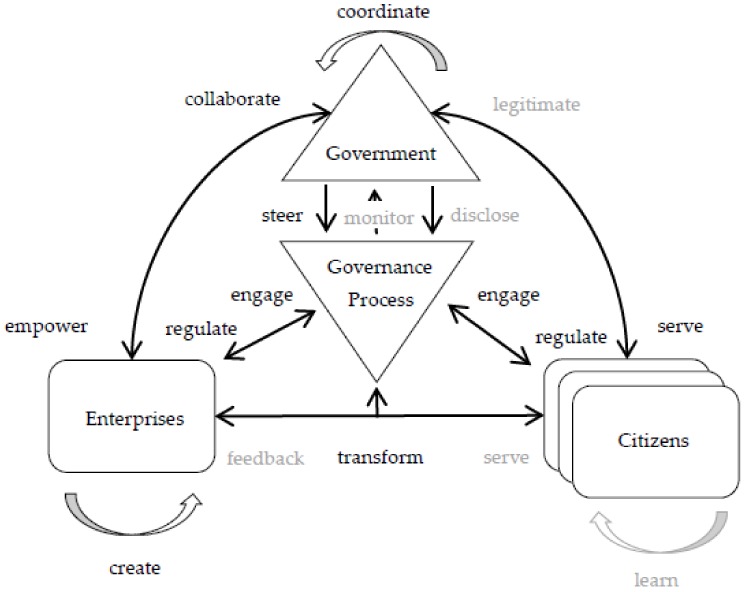
Framework instantiation, Guizhou-cloud big data project case.

The instantiation of the framework in this case is depicted in Figure 3. The BDILDLG is represented as *Governments*, whereas citizens and Internet companies are represented as *Citizens* and *Enterprises* respectively. The goals, strategies, and implementation of big data strategy belong to part of the *Governance Process*. The BDILDLG is composed of provincial, municipal and district leaders who jointly exercise the power of policy decision-making, the creation of big data offices under the guidance of multilevel government coordination is part of the *coordinate* relationship. Otherwise, governments implement big data strategy and other policies through the *steer* and *regulate* relationships. The government authorizes enterprises to establish a government information system and IT infrastructure through *empower* relationship. In contrast, enterprises and government-controlled enterprises sign strategic cooperation framework agreement and establish big data industrial clusters through industrial agglomeration to jointly promote economic development, whose performance is represented by the *collaborate* relationship. This collaborate relationship enables enterprises to share ideas, knowledge, and information with government, which further facilitates the development of new technologies through *create* relationship. Along with the establishment of big data centers, governments are likely to provide data cloud services for enterprises and citizens through the *serve* relationship. The government gets involved in enterprises and targets transformation strategies at them through *transform* relationship.

### 4.3. Case 3—The Zhejiang Internet Plus Government Service Project

#### 4.3.1. Overview of Project

In 2013, Zhejiang province implemented the “Internet+” Government Service Project (IGSP), which consists of two stages. The first stage is represented as a province-wide process re-engineering of public services, named Administrative Examination and Approval System Reform. In early 2014, each city, district, and county in Zhejiang province established the Administrative Examination and Approval Bureaus, mainly responsible for simplification, standardization, and redesign of the process of public services. This act took the initiative to integrate different levels of government and bureaus, as they previously operated rather independently from each other. In this stage, process-driven organization change was implemented. This action is represented by the establishment of the offline one-stop service center, enabling citizens to access all services at one site by physically aggregating government units at all levels. The second stage of IGSP was represented as the establishment of a one-stop service website based on third-party cooperation. The one-stop service website project operates under a strategic partnership agreement initiated in June 2015 between Zhejiang province and one of the largest IT companies in China, Alibaba Group, which is mainly engaged in the digitization of redesigned service process, the development of provincial-level government service website as well as the public-related IT infrastructure of Zhejiang province authority. In 2016, Zhejiang Government Service Website (ZGSW) was officially launched, containing 9000 services of 11 cities, 90 counties and 43 provincial departments. The ZGSW is a centralized platform for delivering e-services to citizens. In other words, the ZGSW is a single interface access to e-services and information offered by different public authorities, which can be employed by citizens to request services from various government departments online. In addition, a unified payment function was included in the ZGSW.

#### 4.3.2. Framework Instantiation

**Figure 4:**
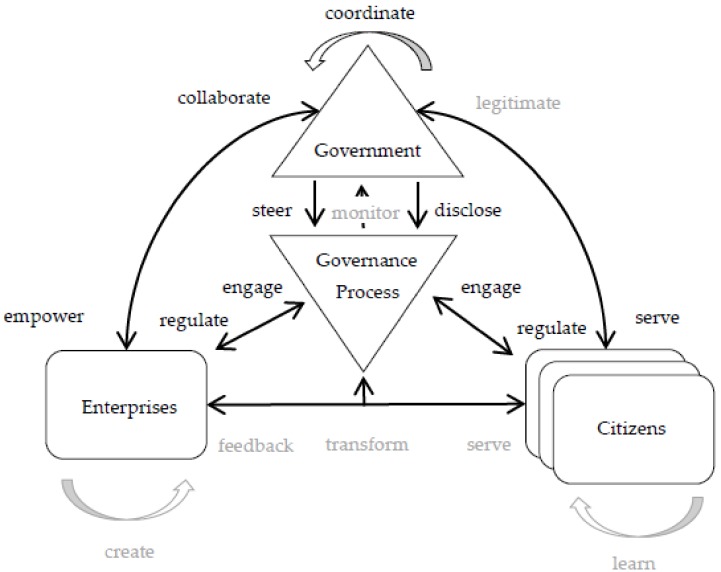
Framework instantiation, Zhejiang Internet plus government service project case.

The instantiation of the framework in this case is depicted in Figure 4. Government agencies are represented as *Governments* whereas Alibaba Group and citizens are represented as *Enterprises* and *Citizens* respectively. The government-led “Internet+” Government Service project is part of the *Governance Process*, including laws and policies related to the development of process reengineering, organizational change and “Internet+” technology embedding. Created by government through the *steer* relationship. The enabling role of the government concerning the provision of online or offline services is achieved through *serve* relationships. Policies implemented through interactions between policymakers, enterprises, and citizens are represented as *regulate* relationships. Furthermore, the open role is played through the *disclose* relationships, and multi-level interaction within the government is represented as *coordinate* relationships. Thanks to the collaboration of Internet enterprises in the “Internet+” Government Service, citizens, and other enterprises make contributions by informing the governance process (*engage*) and sharing their resources with each other.

### 4.4. Case 4—The Guangdong E-Governance Project

#### 4.4.1. Overview of Project

The Guangdong e-governance project operates under a strategic partnership agreement that started in February 2015 between Guangdong province and one of the largest IT companies in China, Tencent Holdings Limited. According to this agreement, Tencent is required to assist the Guangdong province and its subordinated units (such as Guangdong Police Department, Guangdong Communications Department, and Guangdong education bureau) to manage public services on Tencent’s major social media platform, WeChat. Tencent was authorized by the government to set up a public account for each government department to publicize information and deliver public e-services. During this process, the government undertakes the due obligations of providing the content, replying to citizen messages and connecting internal systems with the WeChat platform. In order to further replace the traditional provincial-level service delivery platform to reduce administrative costs, in June 2015, Guangdong province along with its subordinated units signed a new strategic cooperation agreement framework with Tencent to co-develop a digital public service provision platform on WeChat, named the City Services Platform. This platform invites any public sector that delivers e-services to apply from Tencent, which means that the official public service delivery platform embedded with third-party social software platforms in Guangdong province will gradually take the place of official platforms. According to the agreement, Guangdong and the relevant departments are supposed to reorganize the public service processes. In consequence, these government departments began to connect with each other under Tencent’s IT structure, forming an umbrella public service network centered on the City Services Platform. Tencent provides technical support to these government departments to develop customized digital service functions. In reverse, each government department needs to redistribute human and physical resources to the City Services Platform for real-time development and update of the digital service functionality on WeChat, which costs less and has a significantly larger user base. In November 2015, Tencent launched the first version of Guangdong’s City Services Platform, containing an average of 33 functionalities and 457 services.

#### 4.4.2. Framework Instantiation

**Figure 5:**
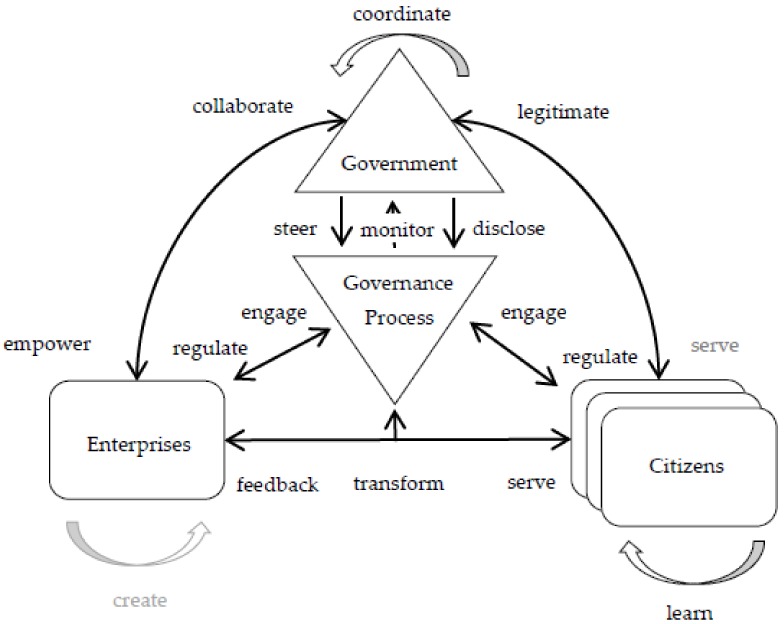
Framework instantiation, Guangdong e-governance project case.

The instantiation of the framework in this case is depicted in Figure 5. Guangdong province and its subordinated units are represented as *Governments* whereas Tencent is represented as *Enterprises*. Citizens and other entities participating in the e-governance project are represented as *Citizens*. The integration of enterprise-led e-service through the City Services Platform is part of *Governance Process*. Government-led e-integration based on WeChat is part of the *coordinate* relationship. Moreover, citizen contributions and Tencent’s technical support belong to the *engage* relationship, which enables citizens to change roles (*learn*) as well as sharing opinions, discussions, and knowledge between themselves and with government via the governance process. Enterprises serve (*serve*) as intermediaries which replace the government in providing public services to citizens through the *collaborate* relationship. The government adopts the governance process via the *steer* relationship and discloses information to citizens via the *disclose* relationship. The governance process speeds up the transformation of e-governance through the *transform* relationship. In turn, enterprises and citizens take advantage of the governance process to *monitor* government operation, which hence is conducive to empowering enterprises to deliver public services and gain trust so as to legitimize *(legitimize*) the government to act on their behalf.

## 5. Cross-Case Analysis and Discussion

### 5.1. Comparison of Government–Enterprise Relations Under Holistic Governance

This section carries out a cross-case analysis of the four cases developed in Section 4 concerning the instantiation of the *Holistic Governance for Sustainable Public Services Framework*. Table 3 and Table 4 summarize the comparison of the four cases from many relevant dimensions.

As shown in Table 3, the four cases cover the entire spectrum of 14 government–enterprise relationships introduced. The case study with the largest number of 13, or 92%, of the relationships is Case 4, followed by Case 1 and Case 2 with 9 or 64% of the relationships respectively. Subsequently, Case 3 has 8 or 57% of the relationships. The overall coverage of the Governments, Enterprises, Citizens and Governance Process entities by the case studies is four instances or 100%. The government-enterprise regulates and serves with 4 or 100% of the instantiations, followed by coordinate, disclose and transform with 3 or 75% of the instantiations, respectively, and followed by learn with 2 or 50% of the instantiations. The relationships with the least number of instantiations are legitimate and monitor which have 1 or 25% of the instantiations.

### 5.2. Comparison Analysis of Characteristics of Holistic Governance Processes

On the basis of Table 3, the four case studies cover all entities and relationships introduced by the holistic Governance for Sustainable Public Service framework. Moreover, these cases differ in the characteristics of holistic governance processes. According to Table 4, the article further compares the four cases by right of the seven-stage model of the public service cycle to identify which characteristics give rise to the differences of holistic governance processes of the four cases.

Firstly, our results are consistent with existing literature on government–enterprise relations in the digital government context. The results show that enterprises are more likely to participate in holistic governance when local governments are constrained by knowledge resources such as technology and talents in China. The relationship between government and enterprise in the whole governance paradigm shows a significant difference in distribution when enterprises participate in different stages of public service supply. We further show that when capabilities include some basic service skills such as information disclosure or digital application development in the design and planning stages of public service delivery, they approach these skills through a combination of empowerment, regulatory, and coordinating relationships in a government-led way. In this manner, the holistic motivation of the project mainly lies in fitting in technological change through the expansion of the cooperation network. An example can be found in case 1, where because mainstream social software has been embedded on the State Council App, policymakers now have the ability to release policy information effectively, which can be ascribed to the information leverage effect of social media. Another example can be found in case 2, where because of the embedding of Alibaba’s big data framework on government data platforms, policymakers currently are able to attract government departments and NGOs to share data. Another finding from these cases is that when capabilities include some basic service skills such as one-stop service or privatizing online service in the execution stages of public service delivery, they approach these skills through a combination of collaborate, transform and coordinate relationships in a complementary way. In this way, the holistic motivation of the project primarily consists of achieving top-down organizational reengineering by virtue of public-private partnerships. In the case 3, due to the aggregation of government offline service platform and enterprise online service platform, policymakers are capable of facilitating the information architecture of enterprises to cooperate with enterprises to provide public services, so as to establish a panoramic public service delivery system. Moreover, suppose that capabilities consist of some basic service skills such as mobile participation or self-service in the output stages of public service delivery, they approach these skills through a combination of coordination, feedback and serve relationships in an enterprise-led way. By this means, the holistic motivation of the project is mainly to reduce administrative costs and address organizational fragmentation through outsourcing public services. As for case 4, because of the provision of public services on the WeChat platform, policymakers can redesign public services from the bottom up according to the WeChat information framework. The outsourcing of public services leads to the reform of the public service system and the reduction in administrative costs.

Secondly, trust-building between governments and enterprises played a pivotal role in nurturing the holistic governance paradigm. Moreover, it also indicates the new role played by Internet companies in digital service provision in terms of pushing the boundaries of the status quo to shift the support for public services beyond administrative governing to holistic governance. Additionally, these Internet companies, serving as political entrepreneurs in the community, can facilitate political innovation and transform the way in which public e-services are provided. For example, Alibaba Group, an Internet company created by Jack Ma in 1999 in Zhejiang province, plays a transformative role in the creation, design, and management of e-commerce in China, also participating in China’s national “One Belt and One Road” strategy relying on “Internet +” technology advantages. More specifically, the founders, not satisfied with just e-commerce platform from the start of the organization, have been searching for creative solutions to transform and provide additional public spaces, with political entrepreneurs crossing and blurring the boundaries of traditional public and private sectors in public services.

Thirdly, one area where all four cases were successful was recognizing the need to tailor the distribution of government-enterprise relationship to the digital needs of the urban areas. Although some core competencies such as mobilization, and platformization are universal and can be obtained in any technical cooperation project with enterprises, there are important differences between the partnership and policy arrangement of holistic governance that requires local adaptations of these generic competencies. For example, Guizhou province that develops big data industrial clusters may long for the participation and coordination of the central government to obtain policy and financial support. Simultaneously, it conceives of using WeChat for free as a service supply platform, which may require equal collaboration with enterprises rather than empowerment or regulation. What is worth mentioning is that using a framework can be useful to ensure coverage of each holistic need, but promoting a holistic governance framework application effectively also requires understanding the partnership.

## 6. Conclusions

The governance of digital public service projects is a complex socio-technical phenomenon, and governments facing a multitude of social, economic, cultural and other policy challenges are supposed to work through co-production with diverse actors to integrate existing resources, knowledge, and skills in the course of seeking sustainable public services. According to the goals, the policy adopted to achieve these goals as well as the context where this policy is implemented, holistic governance occurs in many variations. In particular, it is explicit that great changes have taken place in the reshaping of government–enterprise relationships that are part of holistic governance, and that the conceptual framework identifies a comprehensive set of relationships that account for how decisions by governments or enterprises and mutually accepted governance arrangement dedicate to shaping such relationships and enhancing individual and collective capacity for pursuing sustainable public services. Further case studies show that the supply process of public service moving from administrative governing to holistic governance seems not to be automatic, which requires meeting the demand of local governments and citizens, and extensive resource inputs from enterprises themselves. Our findings show that the interactive relationships between governments and enterprises seem to follow different patterns when enterprises are involved in different stages of public service delivery. To be more specific, resource constraints issued by the government open the window of opportunity for an enterprise’s involvement in the planning and implementation of public service policies. Theoretically, enterprises’ involvement in holistic governance further complicates the relationships between principals and agents existing in various contractual relationships between governments and enterprises. In short, the holistic governance regime, enterprises and governments get involved in joint decision-making in terms of some key aspects of public services delivery.

We can draw three general conclusions. First, it is found that economic incentives and public service strategy need to be developed in combination to inspire implementation and promotion of holistic governance arrangements, as by no means is aware of what holistic governance and its benefits consist of sufficient to promote implementation. Besides, the implementation of holistic governance seems to be more effective when complemented with organizational strategy pertaining to process reengineering and organizational transformation. It is expected that the scope of the holistic governance framework should potentially be expanded to incorporate corresponding structural attributes on how to structure the organization adaptively in relation to the transformation of government in the public service system. Finally, it is critical to embed the holistic governance paradigm in the specific urban contexts and tailor the governance process to the particular characteristics, interests, and expectations of different types of local government. In this respect, exploring governments’ needs in e-services projects as well as investigating the correlation between those needs and the different challenges, they are confronted with information that could be attractive to researchers. The analysis has made contributions to research on holistic governance in China’s digital government context to a certain extent.

Drawing on the analysis of four empirical cases, this study provides several contributions to theory and practice on holistic governance in the context of China’s digital government. In theory, this article suggests that holistic governance is a distinct type of enterprise support for sustainable public services and we need to develop a better understanding of its processes and results. Our study contributes to the public administration and sustainability literature by bridging such a distinction between the planning and the creation of public services and exploring the conditions under which a government–enterprise relationship reshapes from administrative governing to holistic governance. This article established an analytical framework consisting of four entities—government, enterprise, citizen and governance process—in order to assist China’s policymakers to deconstruct instances of the holistic governance for sustainable public services towards conceptual clarity. In practice, our proposed paradigm of adaptive governance based on an analysis of real-life empirical cases also provides key guidance for public managers engaged in the governance of IT-related project collaboration between government and non-government actors. Our findings suggest that Chinese policymakers in all cases take the initiative to adjust the relationship between governments and enterprises in response to fragmented public service systems in China, especially in IT-related endeavors. It is also suggested by research results that policymakers (1) seek the opportunity to cooperate with enterprises in the light of technology to acquire new knowledge and resources; (2) set up the mechanism that engages players from multiple levels of government and multiple sectors for coordination and deployment of the service system; (3) consciously create, coordinate and maintain a favorable institutional elasticity to ensure the sustainability of holistic governance, which is composed of both intergovernmental and cross-sector cooperation; (4) seek organizational transformation to adapt to evolving public service systems. The theoretical and policy implications of this article are huge as public administration scholarship moves from new public management to new public governance, and public managers are facing extensive challenges in sustaining the sustainability of public services and solving complex socio-technical problems on their own.

As with all studies, ours also has a number of limitations. First, the study is the presence of limited pre-selected cases to test the holistic governance framework through the pre-selected cases that were taken for this particular task. Second, this pre-selection may make the findings less generalizable. While the unique political, cultural, economic, and digital characteristics of China provide an intriguing context to test the framework in which to ascertain the critical dimensions of adaptive governance, such a choice may lead to insufficient explanatory power in other contexts. Third, due to the perspective constraints, the framework covers external relationships but ignores institutional factors in such entities and their impact on such relationships. Moreover, the organizational, cultural and other social resources required for various relationships are not covered. Nevertheless, the value of this study lies in its representative case selection and exhaustive methodology to develop the theory and framework based on existing studies on the topic for better comprehension.

Areas for subsequent studies are highlighted. First, given the limited studies on the service-oriented holistic governance paradigm, subsequent studies are also directed at developing universal and holistic methodologies to assess the holistic governance process. Second, subsequent studies aim to test the applicability of the framework to various national, local or departmental contexts. To capture these changing contexts, subsequent studies should stress the selection of case studies. By retaining and reintroducing a certain contextual factor, such processing can provide a unique natural experimental setting to verify the link between certain contextual factors and the configuration of government–enterprise relationships toward the holistic governance paradigm. This also reveals that the cases should be studied in a longitudinal manner to observe the evolution of this paradigm. Third, subsequent studies can primarily investigate how the institutional arrangement of the holistic governance paradigm is recognized by the recipients of public services. For instance, understanding how the holistic governance arrangements impact citizens’ perception of the legitimacy of public service systems will be critical to verify the adaptability and sustainability of each governance arrangement.

## Figures and Tables

**Table 1:** Summary and definition of government–enterprise relationships.

Relationships	Definition	Source
Coordinate	The behavioral consistency of public actors to deal with complex things at various jurisdictional levels.	[49,50]
Collaborate	Government cooperate with enterprises and share and exchange the access to sources.	[51]
Empower	Governments create conditions for enterprises to take up decisions and actions by themselves.	[52]
Legitimate	Citizens and enterprises legitimize governments to act on their behalf.	[53]
Steer	Governments guide citizens and enterprises through various policy instrument.	[54]
Disclose	Governments open their decisions and operations to public scrutiny.	[55]
Monitor	Enterprises and citizens provide real-time supervision and accountability to governments through measurable policy evaluation system.	[56]
Engage	Governments engage enterprises and citizens in co-deciding public policies.	[57]
Transform	Governments achieve structural change and restructuring through adaptive learning.	[58]
Regulate	Governments restrict the conduct of enterprises and citizens to achieve policy objectives.	[59]
Feedback	Citizens report the service experience to the service provider.	[60]
Serve	In order to meet governance demand, governments establish a supply and demand relationship of public goods.	[61,62]
Create	Under the authority of the government, citizens and enterprises create public value for themselves and the community.	[63]
Learn	Bottom-up innovation and co-evolution of self-organized networks of organizations.	[64]

**Table 2:** Analyzed case studies.

Id	Case	Main Resources
Case 1	China state council APP project	http://www.gov.cn/xinwen/2017-02/09/content_5166775.htm
Case 2	The Guizhou-cloud big data project	https://www.gzdata.com.cn/
Case 3	Internet plus government project	http://zwgk.gd.gov.cn/006939748/201604/t20160405_650520.html
Case 4	The Guangdong e-governance project	http://www.echinagov.com/news/256550.htm

**Table 3:** Comparative analysis of distribution of government–enterprise relations.

ID	Entities				Relationships														
	Governments	Enterprises	Citizens	Governance Process	Coordinate	Collaborate	Empower	Legitimate	Steer	Disclose	Monitor	Engage	Transform	Regulate	Feedback	Serve	Create	Learn	Coverage (%)
					1	2	3	4	5	6	7	8	9	10	11	12	13	14	
Case 1	X	X	X	X		X	X		X	X		X	X	X		X		X	64
Case 2	X	X	X	X	X	X	X		X			X	X	X		X	X		64
Case 3	X	X	X	X	X	X	X		X	X		X		X		X			57
Case 4	X	X	X	X	X	X	X	X	X	X	X	X	X	X	X	X		X	92
Coverage (%)					75	100	100	25	100	75	25	100	75	100	25	100	25	50	

**Table 4:** Overview of the characteristics of the four cases.

Characteristics	Case 1	Case 2	Case 3	Case 4
Number of governments	4+	14+	8+	6+
Number of non-governmental organizations (NGOs)	10+	1	2	1
Duration	9 years+	2 years	2 years	2 years
Context	Digitalization	Digitalization	Digitalization	Transformation
Design	Application development based on information and communication technology (ICT)	Application development and development based on big data	Website development based on “Internet+”	Platform design based on WeChat
Motivation of holistic	Technological change	Technological change	Technological change	Process fragmentation
	Insufficient participation		Process fragmentation	Financial deficit
	Information disclosure		Administrative opacity	Poor user experience
		Organization fragmentation economically backward		
Collaborate initiatives	Expanding network of partnerships	Cooperating with enterprises Developing big data industry	Standardizing process	Process reengineering
			Public-private partnership	Outsourcing online service
Service patterns	Government-led	Government-led	Complementary	Enterprise-led
outputs	China State Council App	Guizhou-cloud App	Zhejiang Government Service Website	Guangdong’s City Services Platform
Skills covered from holistic Framework	Participation Coordination	Participation	Participation	Coordination
		Coordination	System integration	Mobile participation
		Data sharing	One-stop service	Service outsourcing
		Big data analysis	Service marketization	Organizational restructuring
